# Preoperative neutrophil-to-lymphocyte ratio is an independent prognostic marker in patients with laryngeal squamous cell carcinoma

**DOI:** 10.1186/s12885-015-1727-6

**Published:** 2015-10-20

**Authors:** Xiu-Ping Tu, Qian-Hui Qiu, Liang-Si Chen, Xiao-Ning Luo, Zhong-Ming Lu, Si-Yi Zhang, Shao-Hua Chen

**Affiliations:** Department of Otorhinolaryngology, Guangdong General Hospital & Guangdong Academy of Medical Sciences, Guangzhou, Guangdong 510080 P. R. China

**Keywords:** Laryngeal squamous cell carcinoma, Neutrophil-lymphocyte ratio, Prognostic marker

## Abstract

**Background:**

Neutrophil-lymphocyte ratio (NLR) has been shown to be associated with prognosis in various solid tumors. This study aimed to evaluate the prognostic role of NLR in patients with laryngeal squamous cell carcinoma (LSCC).

**Methods:**

A total of 141 LSCC patients were retrospectively reviewed. Patients’ demographics were analyzed along with clinical and pathologic data. The optimal cutoff value of NLR was determined using receiver operating characteristic (ROC) curve analysis. The impact of the NLR and other potential prognostic factors on disease-free survival (DFS) and overall survival (OS) was assessed using the Kaplan-Meier method and multivariate Cox regression analysis.

**Results:**

The optimal cutoff value of the NLR was 2.17. In the NLR ≤ 2.17 group, the 1-, 3-, and 5-year DFS rates were 88.2, 73.9 and 69.1 %, respectively, while in the NLR > 2.17 group, the DFS rates were 83.0, 54.6 and 49.2 %, respectively. Correspondingly, the 1-, 3-, and 5-year OS rates were 98.9, 85.1 and 77.4 % in the NLR ≤ 2.17 group and 97.9, 63.8 and 53.3 % in the NLR > 2.17 group, respectively. The multivariate Cox proportional hazard model analysis showed that NLR > 2.17 was a prognostic factor for both DFS [hazard ratio (HR) = 1.869; 95 % confidence interval (CI) 1.078–3.243; *P* = 0.026] and OS (HR =2.177; 95 % CI 1.208–3.924; *P* = 0.010).

**Conclusion:**

Our results showed that elevated preoperative NLR was an independent predictor of poor prognosis for patients with LSCC after surgical resection.

## Background

Laryngeal squamous cell carcinoma (LSCC) is one of the most common head and neck malignancies that remain as significant cause of morbidity and mortality. Approximately 650,000 new cases and 350,000 cancer deaths attributed to this condition occur every year [1, 2]. Treatment options in LSCC include surgery, radiotherapy, chemotherapy, or a combination of surgery, radio- and chemotherapy [3–7]. Despite improvements in treatment, an epidemiological survey indicated that the clinical outcome for LSCC has improved little in the past 20 years [8]. Therefore, it is of great significance to find novel and effective biomarkers for prediction of LSCC patients with poor prognosis or at high risk of early recurrence.

Recently, there is increasing evidence that the host inflammatory response to cancer is associated with poor tumor-specific prognosis. Kawata et al*.* reported that lymphocyte infiltration around the tumor is associated with a better prognosis of HCC [9], whereas neutrophil in tumor stroma is associated with a poor prognosis [10]. Likewise, the neutrophil-to-lymphocyte ratio (NLR), a predictor of the patient’s inflammatory status, has been shown to be an effective prognostic marker in many solid tumors [11–17]. Kum et al*.* showed that neutrophil-to-lymphocyte ratio elevated in squamous cell carcinoma of larynx compared to benign and precancerous laryngeal lesions [18]. To our knowledge, the prognostic value of NLR in laryngeal squamous cell carcinoma has not been reported.

In this study, we evaluated the prognostic value of preoperative NLR in patients with LSCC after surgical resection.

## Methods

### Patients

Clinical data were collected from patients with LSCC who underwent surgical resection at the Department of Otorhinolaryngology, Guangdong General Hospital & Guangdong Academy of Medical Sciences from January 2006 to August 2011. Clinical stage of laryngeal cancer was determined according to the AJCC- TNM stage (7^th^ ed, 2010) [19]. The selective criteria for the patients were as follows: (1) laryngeal squamous cell carcinoma confirmed by pathology, (2) older than 18 years of age; (3) complete clinical, laboratory, imaging, and follow-up data; (4) no evidence of sepsis [11]; (5) no hematological disorders or treatment that could result in an elevated NLR, for example, administration of hematopoietic agents such as granulocyte-colony stimulating factor (G-CSF) within 1 month before surgery; (6) no autoimmune disease or treatment with steroids; (7) no pre-operative treatments such as radiotherapy or chemotherapy; (8) patients were treated with curative intent. Routine blood tests were performed on the day before surgery. NLR was defined as the absolute neutrophil count divided by the absolute lymphocyte count. Patients were followed-up every 3 months for survival status, disease progression and time of death. The last follow-up was 30 September 2014. Disease-free survival (DFS) was recorded from the date of surgery to the date of recurrence, or last follow-up. Overall survival (OS) was recorded from the date of surgery to the date of death or last follow-up. This study was approved by the Research Ethics Committee of Guangdong General Hospital & Guangdong Academy of Medical Sciences. All participants signed informed consent to participate in the study.

### Statistical analysis

All statistical analyses were performed with SPSS version 19.0 software (Chicago, IL, USA). The chi-square test was used to compare categorical variables. Survival curves were plotted using the Kaplan-Meier method and compared using the log-rank test. Factors analyzed by univariate analysis with *P* < 0.05 were included in multiple Cox proportional hazards model. *P* < 0.05 was considered significant.

## Results

### Demographic data

A total of 141 patients were eligible for the study. The median age at diagnosis was 59 (range 36–87) years. There were 137 men and 4 women. Eighty-three patients (58.9 %, 83/141) had a smoking index of 20 pack-year or more [20], and 24 patients (17.0 %, 24/141) had a drinking index of 1000 g-year or more [21]. Surgical procedures included total laryngectomy (43 cases, 30.5 %), partial laryngectomy (64 cases, 45.4 %), and CO2 laser surgery (34 cases, 24.1 %). The locations of tumors included 80.2 % (113/141) glottic, 17.0 % (24/141) supraglottic and 2.8 % (4/141) subglottic. Most of the patients were in the N0 stage (83.7 %, 118/141), with 23 patients (16.3 %, 23/141) in the N1 to N3 stages. Eighty-six patients (61.0 %, 86/141) were in T1 to T2 stages, and 55 patients (39.0 %, 55/141) were in the T3 to T4 stages. Details of features are shown in Table 1.

**Table 1:** Correlation between peripheral NLR and clinicopathologic variables of LSCC patients

		NLR		
Variables	Cases	NLR ≤ 2.17	NLR > 2.17	*P*
Age (years)				
≤ 60	74	48 (64.9 %)	26 (35.1 %)	
> 60	67	46 (68.7 %)	21 (31.3 %)	0.633
Sex				
Male	137	90 (65.7 %)	47 (34.3 %)	
Female	4	4 (100.0 %)	0	0.151
Smoking history				
Yes	83	54 (65.1 %)	29 (34.9 %)	
No	58	40 (69.0 %)	18 (31.0 %)	0.628
Drinking history				
Yes	24	15 (62.5 %)	9 (37.5 %)	
No	117	79 (67.5 %)	38 (32.5 %)	0.635
T classification				
T1–T2	86	62 (72.1 %)	24 (27.9 %)	
T3–T4	55	32 (58.2 %)	23 (41.2 %)	0.087
Lymph node metastasis				
Negative	118	80 (67.8 %)	38 (32.2 %)	
Positive	23	14 (60.9 %)	9 (39.1 %)	0.519
Histological grade				
Well	48	34 (70.8 %)	14 (29.2 %)	
Moderately	73	48 (65.8 %)	25 (34.2 %)	
Poorly	20	12 (60.0 %)	8 (40.0 %)	0.670
Primary location				
Supraglottic	24	15 (62.5 %)	9 (37.5 %)	
Glottic	113	77 (68.1 %)	36 (31.9 %)	0.671
Subglottic	4	2 (50.0 %)	2 (50.0 %)	
Clinical stage				
I–II	80	58 (72.5 %)	22 (27.5 %)	
III–IV	61	36 (59.0 %)	25 (41.0 %)	0.092

*NLR* neutrophil-to-lymphocyte ratio, *LSCC* Laryngeal squamous cell carcinoma

### An optimal cutoff value for elevated NLR

**Fig. 1:**
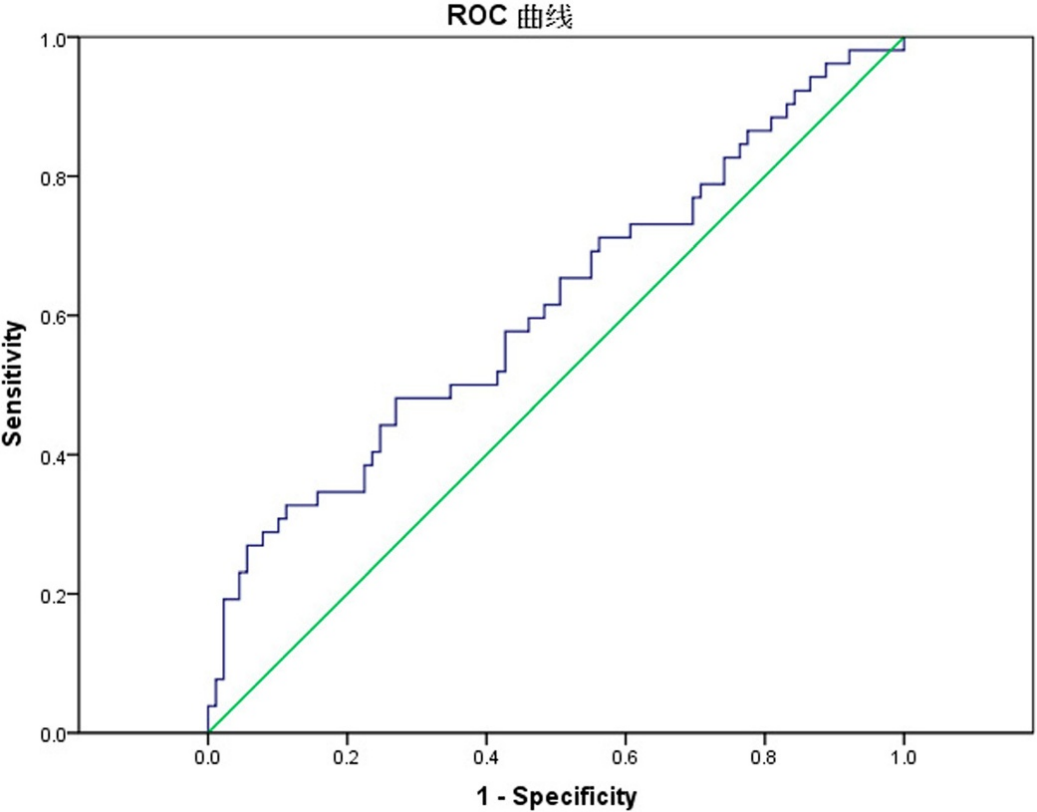
Receiver operating characteristic curves of preoperative neutrophil-to-lymphocyte ratio (NLR) for predicting tumor recurrence in patients with Laryngeal squamous cell carcinoma (LSCC) after surgical resection

To avoid a predetermined cutoff point, the optimal cutoff score of preoperative NLR was defined by receiver operating characteristic (ROC) curve analysis. The cutoff value was that point closest to both maximum sensitivity and specificity. According to the ROC curve, the optimal cutoff value of preoperative NLR was 2.17. The area under the ROC curves was 0.614 with a 95 % confidence interval (95 % CI) for the area between 0.515 and 0.713 (*P* = 0.024) (Fig. 1). Clinicopathological features according to NLR groups are summarized in Table 1.

### DFS and OS according to NLR status

**Fig. 2:**
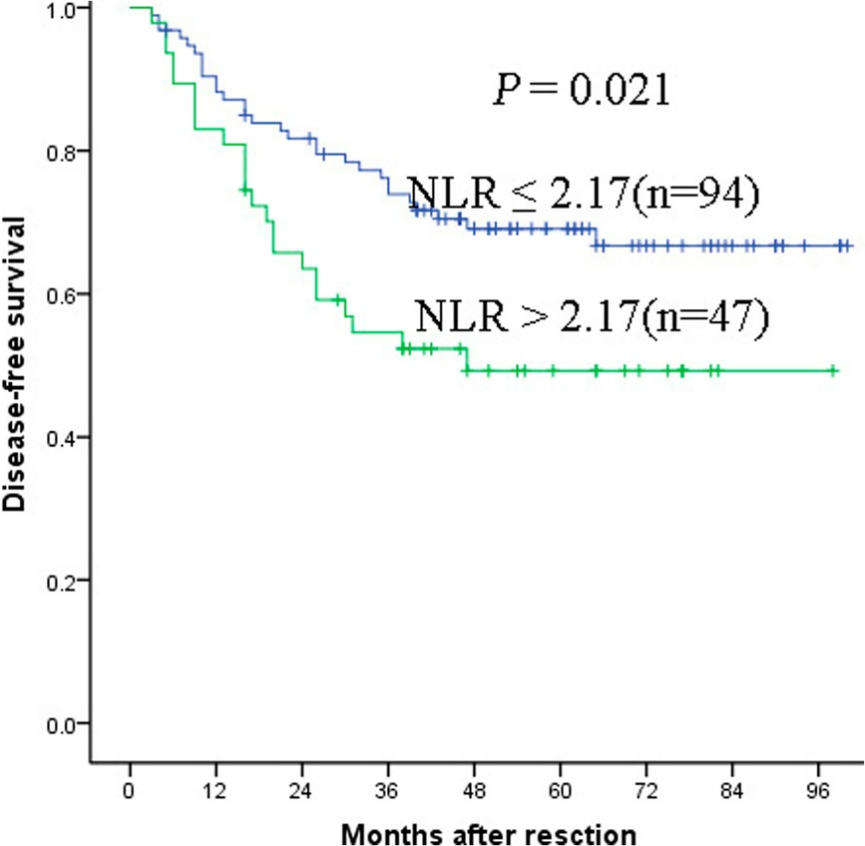
Kaplan-Meier survival curves for DFS in patients with LSCC after surgical resection. Disease-free survival of patients with NLR > 2.17 was shorter than those with NLR ≤ 2.17 (*P* = 0.021, log-rank)

**Fig. 3:**
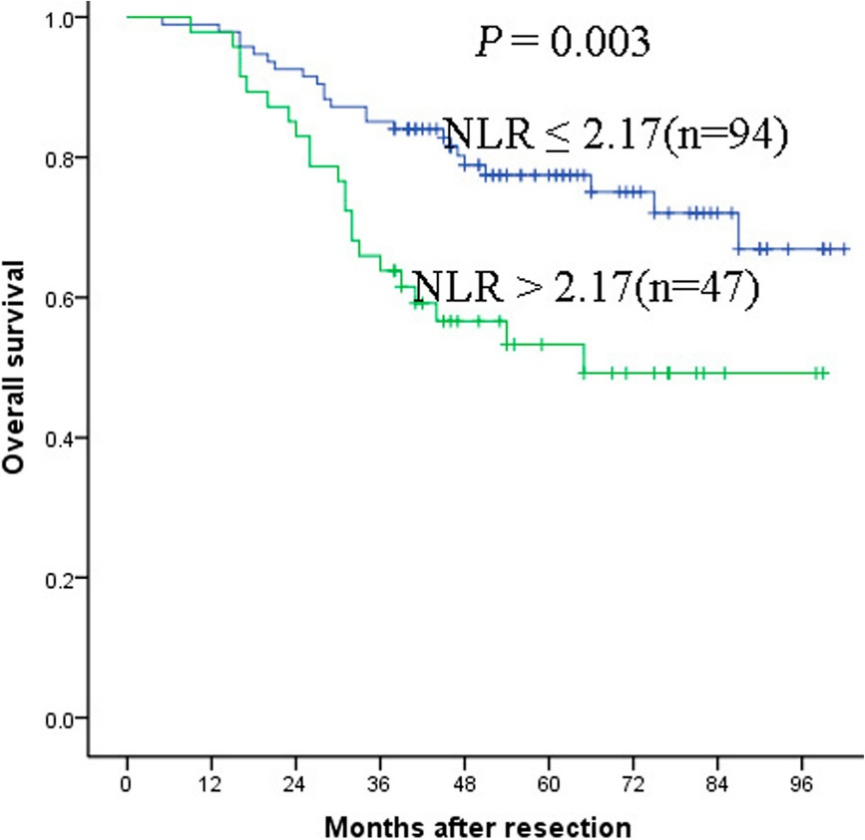
Kaplan-Meier survival curves for OS in patients with LSCC after surgical resection. Overall survival of patients with NLR > 2.17 was also shorter than those with NLR ≤ 2.17 (*P* = 0.003, log-rank)

The 141 patients were divided into two groups: NLR ≤ 2.17 and NLR > 2.17. The median patients’ follow-up was 51 months (range: 5–102 months; mean ± SD: 54.1 ± 23.4 months). Ninety-six patients were alive at the end of follow-up and 52 patients developed recurrence 5–102 months after surgery (median 46 months). In the NLR ≤ 2.17 group, the 1-, 3-, and 5-year DFS rates were 88.2, 73.9 and 69.1 %, respectively, while in the NLR > 2.17 group, the DFS rates were 83.0, 54.6 and 49.2 %, respectively (Fig. 2). Correspondingly, the 1-, 3-, and 5-year OS rates were 98.9, 85.1 and 77.4 % in the NLR ≤ 2.17 group and 97.9, 63.8 and 53.3 % in the NLR > 2.17 group, respectively (Fig. 3). Both DFS and OS of patients in the NLR ≤ 2.17 group were significantly longer than for patients in the NLR > 2.17 group.

### Risk factors for prognosis of LSCC

For all patients included in this study, the 1-, 3- and 5-year DFS rates were 86.5, 67.5 and 62.4 %, respectively. Rates for OS at the 1-, 3- and 5-year period were 98.6, 78.0 and 69.4 %, respectively. Univariate analysis revealed that advanced T classification (T3 and T4 stages), Lymph node metastasis, primary location, NLR > 2.17 and advanced clinic stage were associated with significantly poor DFS. Similarly, advanced T classification, Lymph node metastasis, primary location, NLR > 2.17, poor histological grade and advanced clinical stage all predicted poor OS (Table 2).

**Table 2:** Prognostic factors for DFS and OS by univariate analysis

Variables	*n*	DFS			OS		
		3-years	5-years	*P*	*3*-years	5-years	*P*
Age (years)							
≤ 60	74	65.6 %	64.1 %		75.7 %	72.7 %	
> 60	67	69.4 %	60.7 %	0.980	80.6 %	66.9 %	0.41
Smoking history							
Yes	83	68.3 %	62.7 %		74.7 %	67.2 %	
No	58	66.3 %	62.2 %	0.886	82.8 %	73.0 %	0.464
Drinking history							
Yes	24	66.4 %	61.7 %		66.7 %	61.1 %	
No	117	67.7 %	62.6 %	0.845	80.3 %	71.1 %	0.414
T classification							
T1–T2	86	74.3 %	68.6 %		87.2 %	79.6 %	
T3–T4	55	56.1 %	51.8 %	0.039	63.6 %	53.7 %	0.003
Lymph node metastasis							
Negative	118	73.3 %	68.4 %		83.9 %	75.9 %	
Positive	23	35.5 %	28.4 %	<0.001	47.8 %	35.9 %	<0.001
NLR							
≤ 2.17	94	73.9 %	69.1 %		85.1 %	77.4 %	
> 2.17	47	54.6 %	49.2 %	0.021	63.8 %	53.3 %	0.003
Histological grade							
Well	48	83.0 %	70.5 %		93.7 %	80.4 %	
Moderately	73	59.6 %	59.6 %		74.0 %	67.4 %	
Poorly	20	59.6 %	53.6 %	0.169	55.0 %	49.5 %	0.043
Primary location							
Supraglottic	24	55.9 %	43.1 %		58.3 %	37.4 %	
Glottic	113	69.5 %	66.6 %		82.3 %	77.4 %	
Subglottic	4	75.0 %	50.0 %	0.165	75.0 %	50.0 %	0.001
Clinical stage							
I–II	80	74.9 %	70.3 %		87.5 %	81.1 %	
III–IV	61	57.2 %	51.4 %	0.022	65.6 %	54.5 %	0.004

*DFS* disease-free survival, *OS* overall survival. Other abbreviations are the same as in Table 1

Clinical stage was first excluded from the multivariable analyses because it was a comprehensive variable, and the other variables were entered stepwise into the multivariable Cox proportional hazard model by the forward conditional method. The analysis showed that NLR > 2.17 (HR = 1.869; 95 % CI 1.078–3.243; *P* = 0.026) and lymph node metastasis (HR = 3.224; 95 % CI 1.751–5.937; *P* < 0.001) were independent prognostic factors for DFS in patients with LSCC. NLR > 2.17 (HR = 2.177; 95 % CI 1.208–3.924; *P* = 0.010), lymph node metastasis (HR = 2.968; 95 % CI 1.548–5.692; *P* = 0.001) and advanced T classification (HR = 1.867; 95 % CI 1.018–3.425; *P* = 0.044) were independent prognostic factors for OS in patients with LSCC (Table 3).

**Table 3:** Prognostic factors for DFS and OS as determined by multivariate Cox proportional hazards regression model

Variables	DFS			OS		
	HR	95 % CI	*P*	HR	95 % CI	*P*
T classification				1.867	1.018–3.425	0.044
Lymph node metastasis	3.224	1.751–5.937	<0.001	2.968	1.548–5.692	0.001
NLR	1.869	1.078–3.243	0.026	2.177	1.208–3.924	0.010

*HR* hazard ratio, *CI* confidence interval. Other abbreviations are the same as in Tables 1 and 2

## Discussion

Recent evidence suggests that the development and progression of cancers is closely associated with inflammation and immunity status. The neutrophil-to-lymphocyte ratio is a simple and effective marker of inflammation and immunity. It reflects the patient’s inflammatory and immunity status. NLR has been shown to be a valuable prognostic marker in patients with some solid tumors in many studies, such as hepatocellular carcinoma [11, 22], gastric cancer [23], soft tissue sarcoma [24], esophageal cancer [17, 25] and breast cancer [26, 27]. However, the cutoff value of NLR is not consistent. In most studies, the cutoff value of NLR has been empirically set. To avoid a predetermined cutoff point, we determined that the optimal cutoff value of NLR was 2.17 for predicting prognosis of patients with LSCC by using ROC curve analysis in this study. To the best of our knowledge, this study is the first to evaluate the prognostic role of NLR in patients with LSCC.

We found that patients with elevated NLR (>2.17) had significantly shorter DFS and OS than those with low NLR (≤2.17) (Figs. 2 and 3, Table 2). The multiple Cox proportional hazard regression analysis showed that NLR > 2.17 was an independent prognostic factor of short DFS and OS in patients with LSCC. The relationship between a high pretreatment NLR and poor prognosis has also been observed in patients with other squamous cell carcinoma [17, 25, 28, 29]. Recently, Kum et al*.* demonstrated that NLR was a useful inflammatory marker to differentiate LSCC patients from benign laryngeal lesion (BLL) and precancerous laryngeal lesion (PLL) patients [18].

Although the association between elevated NLR and poor prognosis has been confirmed by many studies, the underlying mechanisms have not yet been fully elucidated [11–16]. One possible explanation is that a relatively increased number of circulating neutrophils produced and secreted angiogenesis-regulating growth factors, chemokines and proteases (e.g., vascular endothelial growth factor (VEGF) [30], interleukin-8 (IL-8) [31], intercellular adhesion molecule 1 [32] and matrix metalloproteinase (MMP)) [33] which may promote tumor growth and metastasis resulting in poor prognosis. Another explanation is that the host immune response to tumors is lymphocyte dependent. Patients with elevated NLR usually have relative lymphocytopenia, and this may reflect a deficient immune response to tumors mediated by CD4^+^ T-helper and cytotoxic CD8^+^ cell [34].

## Conclusions

This study demonstrated that elevated preoperative NLR was an independent prognostic marker of poor DFS and OS in patients with LSCC. NLR is a simple, easily accessible indicator for identifying LSCC patients following surgical resection. It might help clinicians support intensive follow-up surveillance and adopt personalized adjuvant therapies for patients with LSCC who were at high risk for recurrence after surgical resection. It is noted that this study is limited by its retrospective nature and the relatively small size in a single-center. Further multicenter, large prospective studies are needed to confirm the findings.

## Abbreviations

NLR: Neutrophil-lymphocyte ratio
LSCC: Laryngeal squamous cell carcinoma
ROC: Receive operating characteristic
DFS: Disease-free survival
OS: Overall survival
HR: Hazard ratio
CI: Confidence interval
G-CSF: Granulocyte-colony stimulating factor
BLL: Benign laryngeal lesion
PLL: Precancerous laryngeal lesion
VEGF: Vascular endothelial growth factor
IL-8: Interleukin-8
MMP: Matrix metalloproteinase
